# Bilirubin is independently associated with oxidized LDL levels in young obese patients

**DOI:** 10.1186/1758-5996-7-4

**Published:** 2015-01-23

**Authors:** Henrique Nascimento, Ana Inês Alves, Susana Coimbra, Cristina Catarino, Diana Gomes, Elsa Bronze-da-Rocha, Elísio Costa, Petronila Rocha-Pereira, Luísa Aires, Jorge Mota, Helena Ferreira Mansilha, Carla Rêgo, Alice Santos-Silva, Luís Belo

**Affiliations:** Instituto de Biologia Molecular e Celular (Institute for Molecular and Cell Biology), Universidade do Porto, Porto, Portugal; Instituto de Investigaçãoe Inovação em Saúde (Institute for Research and Innovation in Health), Universidade do Porto, Porto, Portugal; Biological Science Department, Faculty of Pharmacy, University of Porto, Porto, Portugal; CESPU, Institute for Research and Advanced Training in Health Sciences and Technologies, Gandra, Portugal; Health Science Research Centre, University of Beira Interior, Covilhã, Portugal; Research Centre in Physical Activity, Health and Leisure – CIAFEL, Faculty of Sport, University of Porto, Porto, Portugal; High Education Institute from Maia (ISMAI), S. Pedro Avioso, Portugal; Childhood and Adolescence Department/Pediatric Service of Porto Hospital Centre, Porto, Portugal; Children and Adolescent Centre, CUF Hospital. CINTESIS. Faculty of Medicine, University of Porto, Porto, Portugal; Departamento de Ciências Biológicas, Faculdade de Farmácia da Universidade do Porto, Rua de Jorge Viterbo Ferreira, 228, Porto, 4050-313 Portugal

**Keywords:** Bilirubin, Oxidized-LDL, Oxidative stress, Atherosclerosis, Pediatric obesity

## Abstract

**Background:**

Bilirubin can prevent lipid oxidation *in vitro*, but the association *in vivo* with oxidized low-density lipoprotein (Ox-LDL) levels has been poorly explored. Our aim is to the association of Ox-LDL with total bilirubin (TB) levels and with variables related with metabolic syndrome and inflammation, in young obese individuals.

**Findings:**

125 obese patients (13.4 years; 53.6% females) were studied. TB, lipid profile including Ox-LDL, markers of glucose metabolism, and levels of C-reactive protein (CRP) and adiponectin were determined. Anthropometric data was also collected.

In all patients, Ox-LDL correlated positively with BMI, total cholesterol, LDLc, triglycerides (TG), CRP, glucose, insulin and HOMA_IR_; while inversely with TB and HDLc/Total cholesterol ratio (*P* < 0.05 for all). In multiple linear regression analysis, LDLc, TG, HDLc and TB levels were significantly associated with Ox-LDL (standardized Beta: 0.656, 0.293, −0.283, −0.164, respectively; *P* < 0.01 for all). After removing TG and HDLc from the analysis, HOMA_IR_ was included in the regression model. In this new model, LDLc remained the best predictor of Ox-LDL levels (β = 0.665, *P* < 0.001), followed by TB (β = −0.202, *P* = 0.002) and HOMA_IR_ (β = 0.163, *P* = 0.010).

**Conclusions:**

Lower bilirubin levels may contribute to increased LDL oxidation in obese children and adolescents, predisposing to increased cardiovascular risk.

## Findings

### Introduction

Bilirubin has important antioxidant and anti-inflammatory properties. Actually, bilirubin can protect low-density lipoprotein (LDL) from oxidation [1] and persons expressing Gilbert syndrome (i.e., unconjugated hyperbilirubinemia) present lower circulating levels of oxidized-LDL (Ox-LDL) [2]. Stojanov et al. [3] reported that healthy young men (18 to 22 years old) with high total bilirubin (TB) have lower concentration of Ox-LDL. Furthermore, a protective association between serum bilirubin level and cardiovascular disease (CVD) and death exists in both men and women [4]. Besides, serum bilirubin levels were inversely associated with body mass index (BMI) values in adults [5], and in a study involving American children and adolescents, bilirubin concentrations were found to be inversely associated with metabolic syndrome, which seemed to be related to the insulin resistance (IR) status [6].

Obese children and adolescents already present with altered CVD risk markers, namely higher IR, triglycerides (TG), LDL cholesterol (LDLc) and C-reactive protein (CRP) concentrations and lower high-density lipoprotein cholesterol (HDLc) and adiponectin levels compared to controls [7]. It was also reported that Ox-LDL level is higher in overweight/obese adolescents (compared to lean adolescents) and that obesity and IR are main independent predictors of higher Ox-LDL [8, 9]. However, the association of bilirubin with Ox-LDL levels in young obese patients is unknown.

The aim of our study is to evaluate the association of Ox-LDL with clinical data of young obese patients and several biochemical variables, including total bilirubin, lipid profile, and markers of glucose metabolism.

## Methods and procedures

### Subjects

Obese children and adolescents, aged 11–18 years, were identified from medical records, at the outpatient clinics of pediatric obesity in two hospitals in Oporto - Portugal. A group of children from 5 primary and 2 middle and high public schools from Oporto suburban setting were also recruited to this study, enlarging the obese group.

The study protocol was approved by the Committee on Ethics of both Hospitals and by the Review Committee of the Scientific Board of the Faculty of Sport of the University of Oporto.

A total of 125 obese children and adolescents participated in the study after informed and written consent of their parents. Smokers, subjects with endocrine disorders, inflammatory, hepatic or infectious diseases or under any therapy that could interfere with our results were excluded from the study.

### Procedures and assays

#### Anthropometric characterization and clinical evaluation

All participants were subjected to clinical examination. Height and weight were measured. Obesity was defined as body mass index (BMI) z-score greater than +1.65 for age and gender, according to 2000 Centre for Disease Control and Prevention (CDC) growth charts. Waist circumference was measured at the superior border of the iliac crest, according to the protocol of the NHANES [10]. Waist-to-Height ratio (WtHr) was calculated and a value greater than 0.5 was considered as predictor of cardiometabolic risk.

#### Blood samples

Blood was collected by venipuncture in EDTA containing tubes, after overnight fasting (10-12 h) and processed within 2 h of collection. Aliquots of plasma were made, and immediately stored at −80 °C until assayed.

#### Plasma analysis

Plasma levels of CRP were determined by immunoturbidimetry [CRP (latex) High-Sensitivity, Roche Diagnostics] and quantification of TB was performed by a colorimetric test (diazotized sulfanilic acid reaction, Roche Diagnostics). Adiponectin plasmatic concentration was evaluated by using a standard commercial enzyme-linked immunoassay (Bender MedSystems, San Diego, CA, USA).

The determination of circulating levels of glucose and insulin was performed by using routine automated technology (ABX Diagnostics). Homeostasis model assessment of insulin resistance (HOMA_IR_) was calculated according to Matthews [11].

The lipid profile was performed in an autoanalyzer (Cobas Mira S, Roche, Basel, Switzerland), using commercially available kits; total cholesterol and TG concentrations were determined by enzymatic colorimetric tests (cholesterol oxidase-phenol aminophenazone and glycerol-3-phosphate oxidase-phenol aminophenazone methods, Roche, resp.); HDLc was measured using enzymatic colorimetric test, after selective separation of HDLc fraction (direct HDL cholesterol, Roche); LDLc was calculated using Friedewald formula [12]; Ox-LDL was measured directly in plasma by using a two-site enzyme immunoassay (oxidized LDL ELISA, Mercodia, Uppsala, Sweden).

### Statistical analysis

The distributions of continuous variables were analyzed using Kolmogorov-Smirnov tests to assess significant departures from Normality. Normally distributed variables are presented as mean ± SD (standard deviation) and those non-normally distributed are presented as median (interquartile range). Comparisons between two groups were performed using Student’s unpaired t-test or Mann–Whitney U test. The association between categorical variables was analyzed using chi-squared test and Fisher’s exact test.

The strength of the association between the variables was estimated by Pearson correlation coefficient, after log transformation of the variables (whenever necessary). To evaluate the contribution of the different variables (clinical and biochemical) to Ox-LDL levels, multiple regression analysis was performed, using stepwise selection, with an entry criteria of *P* <0.05. Age, sex, Tanner stage, weight, BMI, BMI z-score, waist circumference, WtHr, TG, total cholesterol, LDLc, HDLc, glucose, insulin, HOMA_IR_, CRP, adiponectin and TB were included in the analysis as independent variables.

Statistical analysis was performed using the Statistical Package for Social Sciences (SPSS, version 21.0) for Windows (SPSS Inc., Armonk, NY, USA). Statistical significance was accepted at *P* less than 0.05.

## Results

The mean age of the participants was 13.4 years old, and only a minority was pre-pubertal (7.2%). The clinical and analytical data of the whole obese group, and according to Ox-LDL levels, are presented in Table 1. The obese patients were divided in two groups on the basis of their Ox-LDL level, using the cut-off of 39.2 U/l, which corresponds to the median value for the entire group. The two groups were matched for age, gender, and number of pre-pubertal participants. The group with higher Ox-LDL (equal or higher than 39.2 U/l) presented significantly higher BMI values, higher levels of TG, total cholesterol, LDLc, and CRP, and significantly lower values of TB and HDLc/Total cholesterol ratio (Table 1). BMI z-score, waist circumference and WtHr values were higher in the group with higher Ox-LDL, but results did not achieve statistical significance.

**Table 1:** **Anthropometric, nutritional and biochemical variables for total obese patients, and according to oxidized-LDL (Ox-LDL) levels (lower or higher than the median value observed for total patients, 39.2 U/l)**

	All obese patients	Ox-LDL < 39.2 U/l	Ox-LDL ≥ 39.2 U/l	
	(***n*** = 125)	(***n*** = 62)	(***n*** = 63)	***P*** ^***a***^
**Age (years)**	13.4 ± 1.9	13.3 ± 1.8	13.6 ± 1.9	0.351
**Sex (females (%))**	67 (53.6)	35 (56.5)	32 (50.8)	0.592
**Tanner (pre-pub (%))**	9 (7.2)	4 (6.5)	5 (7.9)	1.000
**Height (cm)**	161.8 ± 9.0	162.4 ± 9.2	161.3 ± 8.8	0.490
**Weight (Kg)**	89.3 ± 22.4	86.7 ± 18.9	91.9 ± 25.3	0.196
**BMI (kg/m** ^**2**^ **)**	33.7 ± 6.2	32.6 ± 4.9	34.9 ± 7.1	0.041
**BMI z-score**	2.28 ± 0.32	2.24 ± 0.29	2.32 ± 0.36	0.150
**Waist circumference (cm)**	104.9 ± 15.2	103.7 ± 13.3	105.9 ± 16.8	0.420
**WtHr**	0.65 ± 0.08	0.64 ± 0.07	0.66 ± 0.09	0.227
**TG (mg/dl)**	89.0 (60.0-119.5)	66.0 (53.0-98.0)	108.0 (85.0-135.0)	<0.001
**Total cholesterol (mg/dl)**	150.0 (133.0-173.5)	135.5 (117.8-155.3)	163.0 (149.0-187.0)	<0.001
**HDLc (mg/dl)**	40.0 (36.0-48.0)	41.0 (37.0-49.3)	40.0 (35.0-46.0)	0.119
**LDLc (mg/dl)**	89.0 (73.5-107.5)	76.0 (65.0-87.3)	105.0 (90.0-121.0)	<0.001
**HDLc/Total cholesterol**	0.28 (0.24-0.33)	0.33 (0.29-0.36)	0.24 (0.21-0.26)	<0.001
**Ox-LDL (U/l)**	39.2 (32.5-47.6)	32.5 (28.5-36.4)	47.5 (42.9-53.3)	
**Glucose (mg/dl)**	79.0 (74.0-85.5)	78.0 (73.8-84.3)	80.0 (74.0-86.0)	0.233
**Insulin (μU/ml)**	18.6 (13.8-26.9)	17.7 (13.1-23.4)	21.3 (14.4-29.6)	0.149
**HOMA**_**IR**_	3.58 (2.63-5.52)	3.27 (2.56-4.47)	3.91 (2.64-6.36)	0.106
**CRP (mg/l)**	1.83 (0.88-4.48)	1.33 (0.66-3.58)	2.86 (1.04-5.03)	0.004
**Adiponectin (μg/ml)**	5.14 (3.59-7.04)	4.77 (3.38-7.02)	5.28 (3.91-7.12)	0.174
**Total bilirubin (μmol/l)**	10.60 (9.06-15.39)	11.97 (9.92-18.47)	9.92 (7.18-14.02)	0.001

Results are presented mean ± standard deviation or median (interquartile range), unless otherwise indicated.

Pre-pub, pre-pubertal; BMI, body mass index; WtHr, Waist-to-Height ratio; TG, triglycerides; HDLc, high-density lipoprotein-cholesterol; LDLc, low-density lipoprotein-cholesterol; VLDLc, very low-density lipoprotein-cholesterol; Ox-LDL, oxidized low-density lipoprotein; HOMA_IR_, homeostasis model assessment insulin resistance; CRP, C-reactive protein.

^a^Lower Ox-LDL group *versus* higher Ox-LDL group.

No statistically significant differences were found for metabolic health markers between clinical and school-based samples (data not shown).

In all patients, Ox-LDL correlated positively and significantly with BMI (*r* = 0.197, *P* = 0.027), total cholesterol (*r* = 0.630, *P* < 0.001), LDLc (*r* = 0.678, *P* < 0.001), TG (*r* = 0.510, *P* < 0.001), CRP (*r* = 0.224, *P* = 0.012), glucose (*r* = 0.196, *P* = 0.029), insulin (*r* = 0.186, *P* = 0.038) and HOMA_IR_ (*r* = 0.209, *P* = 0.019); moreover, Ox-LDL levels correlated inversely and significantly with TB (*r* = −0.252, *P* = 0.005) and HDLc/Total cholesterol ratio (*r* = −0.732, *P* < 0.001).

In multiple linear regression analysis, LDLc, TG, HDLc and TB levels were statistically associated with Ox-LDL values (Table 2, model 1). Because the associations with TG and HDLc were highly expected, we re-ran multiple linear regression analysis, after exclusion of these two variables (LDLc was kept in the analysis since is the substrate for oxLDL). In this new analysis, LDLc remained the best predictor of Ox-LDL levels, followed by bilirubin and HOMA_IR_ (Table 2, model 2).

**Table 2:** **Main determinants of oxidized LDL (Ox-LDL) levels by multiple linear regression analysis**

Dependent variable	Model	Unstandardized Coefficients		Standardized Coefficients	t	***P***
		B	Std. Error	Beta		
	**Model 1** (R^2^ = 0.672)					
**Ln Ox-LDL**	(Constant)	1.598	0.300		5.322	<0.001
	Ln LDLc	0.624	0.054	0.656	11.582	<0.001
	Ln TG	0.152	0.029	0.293	5.300	<0.001
	Ln HDLc	−0.318	0.061	−0.283	−5.212	<0.001
	Ln Bilirubin	−0.091	0.030	−0.164	−3.100	0.002
	**Model 2** (R^2^ = 0.524)*					
**Ln Ox-LDL**	(Constant)	1.006	0.286		3.522	0.001
	Ln LDLc	0.632	0.059	0.665	10.719	<0.001
	Ln Bilirubin	−0.113	0.035	−0.202	−3.231	0.002
	Ln HOMA_IR_	0.077	0.029	0.163	2.608	0.010

HDLc, high-density lipoprotein-cholesterol; LDLc, low-density lipoprotein-cholesterol; TG, triglycerides; HOMA_IR_, homeostasis model assessment insulin resistance.

*Re-ran of multiple linear regression analysis, after exclusion of two variables (TG and HDLc).

## Discussion

As far as we know this is the first study demonstrating an association of bilirubin with Ox-LDL levels in young obese patients.

The strong positive correlations found between Ox-LDL and both LDLc and TG (with their inclusion in the regression model of Ox-LDL) are in accordance with literature. Ox-LDL level is likely to increase with the number of LDL particles, and high levels of TG lead to the formation of smaller, denser LDL species, which are known to be more prone to oxidative modification [13, 14]. On the other hand, HDL is known as an atheroprotective protein and is capable to protect LDL from oxidative modification *in vitro*[15, 16]. This is in agreement with the inclusion of HDL in our regression analysis of Ox-LDL (β = −0.283, *P* < 0.001). Bilirubin was also an independent predictor of Ox-LDL levels (Table 2, model1). Actually, it was previously demonstrated that bilirubin is also capable to prevent LDL from oxidation *in vitro*[1] and two recent studies involving young males [3] and patients under hemodialysis [17] reported a similar association *in vivo* between bilirubin and Ox-LDL, suggesting a potential cardiovascular risk protection of bilirubin.

In children, Ox-LDL was found to associate with adiposity and, independently of body fatness, with IR [18]. Indeed, previous reports have demonstrated that obesity and IR are main independent predictors of higher Ox-LDL [8, 9]. Ryder *et al.*[9] reported that IR is an independent predictor of Ox-LDL when whole-body insulin sensitivity index is used, but not when HOMA_IR_ is used. Another study found that Ox-LDL is significantly correlated with fasting insulin and HOMA_IR_, but these relationships disappeared after adjustment for BMI [8]. In the present study, BMI and markers of glucose metabolism were positively and significantly correlated with Ox-LDL levels. However, only after removing TG and HDLc from the analysis, HOMA_IR_ was included in the regression model (model 2; Table 2). In this new model, bilirubin was the second best predictor of Ox-LDL levels (β = −0.202, *P* = 0.002), after LDLc (kept in the analysis) and followed by HOMA_IR_ (β = 0.163, *P* = 0.010), explaining approximately 52% of the variance. It is noteworthy that none adiposity marker entered this new model, probably due to adjustment for glucose metabolism markers. Indeed, both insulin and HOMA_IR_ were highly statistically positively correlated with adiposity markers, namely with BMI, BMI z-score, waist circumference and WtHr (*r* > 0.420, *P* < 0.001 for all).

In the present study, bilirubin was included in both regression models, independently of confounders. These results suggest that the antioxidant and/or anti-inflammatory properties of bilirubin may explain *per se* its negative association with Ox-LDL. However, it is also possible that other antioxidant compounds may contribute to this association. That is, bilirubin levels may actually be a marker of the status of the antioxidant system. Besides bilirubin, the endogenous antioxidant system includes other compounds that have a role in the protection from the deleterious effects of oxygen free radicals.

A previous work from our group demonstrated that young obese patients with higher body fat percentage present lower circulating levels of bilirubin and a higher degree of inflammation [19]. In the present study we did not assess body composition (due to logistic reasons) and the only anthropometric marker related with Ox-LDL was BMI. However, it would be interesting to investigate if body fat mass in obese patients relates concomitantly with bilirubin and Ox-LDL. Furthermore, in the present study, the lack of a significant association between waist circumference and Ox-LDL levels contrast with previous findings in lean and overweight/obese adolescents [9] and may indicate that this is not a consistent or sensitive predictor of Ox-LDL amongst different youth populations. Small inter-individual differences in this measurement (performed at different institutions and only in obese patients) may have contributed to the absence of the association but are unlikely to explain this result by itself. Indeed, the correlation between waist circumference and Ox-LDL was far from being statistically significant. In addition, waist circumference presented well known correlations with other variables, such as insulin, HOMA_IR_, TG and HDLc (all statistically significant; data not shown).

Adiponectin is an important antidiabetic and antiatherogenic adipokine. In obese children and adolescents, adiponectin levels are inversely and significantly correlated with BMI, TG, and HDLc/Total cholesterol, and correlated positively and significantly with HDLc [20]. In the present study we found no significant association between adiponectin and Ox-LDL levels, not suggesting a major role of this adipokine in regulating Ox-LDL level in young obese patients.

In conclusion, TB is independently and negatively associated with OX-LDL levels in young obese patients. Thus, patients presenting lower bilirubin may be at increased atherogenic risk and strategies to reduce Ox-LDL levels should be seek early in life.

## Abbreviations

BMI: Body mass index
CRP: C-reactive protein
CVD: Cardiovascular disease
HDL: High density lipoprotein
HDLc: HDL-cholesterol
HOMA_IR_: Homeostasis model assessment of insulin resistance
IR: Insulin resistance
LDL: Low density lipoprotein
LDLc: LDL-cholesterol
Ox-LDL: Oxidized-LDL
TB: Total bilirubin
TG: Triglyceride
WtHr: Waist-to-Height ratio.
